# Adults with reading difficulties demonstrate selective impairments in the fine neural tuning for print

**DOI:** 10.3389/fnins.2025.1520367

**Published:** 2025-02-11

**Authors:** Tongjie Zhuang, Yaowen Li, Yufei Tan, Jiuju Wang, Xiuyue Yue, Licheng Xue, Jing Zhao

**Affiliations:** ^1^Jing Hengyi School of Education, Hangzhou Normal University, Zhejiang, China; ^2^School of Psychology, Northwest Normal University, Lanzhou, China; ^3^School of Psychology, Shenzhen University, Shenzhen, China; ^4^NHC Key Laboratory of Mental Health (Peking University), National Clinical Research Center for Mental Disorders (Peking University Sixth Hospital), Peking University Institute of Mental Health, Peking University Sixth Hospital, Beijing, China; ^5^School of Preschool Education, Hangzhou Polytechnic, Zhejiang, China; ^6^Zhejiang Philosophy and Social Science Laboratory for Research in Early Development and Childcare, Hangzhou Normal University, Zhejiang, China

**Keywords:** reading, neural tuning for print, visual word processing, Chinese characters, N170, reading difficulty

## Abstract

**Introduction:**

Neural tuning for print, reflected in differential responses of the N170 component of event-related potentials to orthographic forms and other visual stimuli, serves as the neural basis for efficient visual word reading. Impaired neural tuning for print is well established in dyslexic children. Although many adults also experience reading difficulties, relatively few studies have examined whether such impairments exist in adults, particularly those who read Chinese, which differs markedly in visual and linguistic characteristics from alphabetic scripts.

**Methods:**

To fill this gap, we assessed 20 high-level and 16 low-level adult readers who were the two extremes of the best and poorest readers of a database, which consisted of 308 college students. Using ERP techniques, we investigated two levels of neural tuning for print: coarse tuning (i.e., real, pseudo, false characters vs. stroke combinations) and fine tuning (i.e., real vs. pseudo vs. false characters).

**Results:**

Results indicated that high-level adult readers exhibited both coarse and fine tuning for print. In contrast, low-level adult readers displayed a stronger N170 response to real and pseudo characters than to stroke combinations, suggesting intact coarse tuning. However, they showed no reliable N170 differences between real, false, and pseudo characters, indicating impaired fine tuning.

**Conclusion:**

These findings suggest a selective impairment in fine tuning for print among Chinese adults with reading difficulties and support the notion of persistent impairment in fine neural tuning for print among poor readers throughout development.

## Introduction

Reading is a crucial means of acquiring knowledge and is essential for human learning, cognitive development, and cultural transmission. However, the National Assessment of Adult Literacy in the United States indicates that 14% of adults struggle to read most printed materials intended for adults, and nearly 29% find it challenging to perform reading tasks beyond a basic level (Kutner et al. 2007). Despite the significant proportion of adults with reading difficulties, research on this population remains relatively scarce (Venezky and Sabatini, 2002). Specifically, the neural basis for difficulties in visual word processing among Chinese adults with reading difficulties is particularly unclear. To address this gap, we examined electrophysiological responses to visual words in Chinese adults with varying reading levels using a grouping design.

Rapid and accurate processing of visual words is fundamental to reading (Ehri and Soffer, 1999; Zhao et al., 2019). Extensive research has established that the neural basis of efficient visual word processing is grounded in neural tuning for print (Schlaggar and McCandliss, 2007). The N170 component of event-related potentials (ERPs), which exhibits selectivity for words relative to other visual stimuli, is a robust electrophysiological indicator of this neural tuning for print (Bentin et al., 1999; Mahé et al., 2013; Maurer et al., 2005; Schendan et al., 1998; Zhao et al., 2014). Specifically, previous studies have shown that adults exhibit both coarse and fine tuning for print. Coarse tuning for print refers to the selectivity for word and word-like stimuli compared to control stimuli, such as words, pseudo words, letter strings versus symbol strings in alphabetic scripts and real, pseudo, or false characters versus stroke combinations in the Chinese writing system (Xue et al., 2023; Zhao et al., 2014). For example, studies have observed a stronger N170 response to words compared to symbol strings (Araújo et al., 2015; Brem et al., 2005; Maurer et al., 2005, 2007) and a stronger N170 response to real, pseudo, or false Chinese characters compared to stroke combinations (Lin et al., 2011; Zhao et al., 2012). Fine tuning for print refers to selectivity among word and word-like stimuli, such as words versus pseudowords versus consonant letter strings in alphabetic scripts and real versus pseudo versus false characters in the Chinese writing system (Xue et al., 2023; Zhao et al., 2014). Research has found that adults show a stronger N170 response to words and pseudowords compared to consonant letter strings (Bacher et al., 1991; Coch and Mitra, 2010; Martin et al., 2006; McCandliss et al., 1997) and a stronger N170 response to words compared to pseudowords (Mahé et al., 2012; Maurer et al., 2005). Similarly, Chinese adult readers exhibit a stronger N170 response to real and pseudo characters compared to false characters (Lin et al., 2011). Neural tuning for print plays a key role both in fast visual word reading in adults and reading development in children (Fraga González et al., 2014; also see Price and Devlin, 2011 for more detailed discussion; Schlaggar and McCandliss, 2007).

Research has consistently found that neural tuning for print is impaired in individuals with developmental dyslexia (DD), a specific learning disorder in reading acquisition despite adequate intelligence, conventional education, and a normal sociocultural context (American Psychiatric Association, 2013; see Amora et al., 2022 for a recent meta-analysis; Araújo et al., 2012; Hasko, 2013; Mahé et al., 2012; Shaywitz et al., 2003). For example, Maurer and colleagues found that second-grade children with DD showed reduced coarse tuning for print compared to typically developing (TD) children during the initial phase of learning to read (Maurer et al., 2005). A longitudinal study from second to fifth grade revealed that by fifth grade, the differences in coarse tuning for print between DD and TD children were no longer significant (Maurer et al., 2011). Similarly, a recent study found that Chinese third-grade children with DD exhibited coarse tuning for print similar to that of TD children (Xue et al., 2023). These findings suggest a developmental delay rather than a persistent deficit in coarse tuning for print among DD children. However, deficits in fine tuning for print persist throughout reading development in DD children. For instance, DD children showed reduced N170 differences between words, pseudowords, and consonant letter strings compared to TD children (Bakos et al., 2018). Likewise, a recent study of Chinese children found that TD children displayed reliable N170 selectivity for pseudo characters relative to false characters, whereas DD children showed no significant N170 difference between pseudo and false characters (Xue et al., 2023).

While extensive research has focused on children with developmental dyslexia (DD), there is increasing evidence examining adults with DD as well. Multiple studies indicate that adults with DD exhibit reduced coarse tuning for print compared to typically developing (TD) adults (Helenius, 1999; Mahé et al., 2012; Shaul et al., 2012), suggesting a deficit in coarse tuning for print in DD adults. Additionally, deficits in fine tuning for print persist in DD adults. For instance, Araújo and his colleagues has found no difference between pseudowords and unpronounceable letter strings in DD adults (Araújo et al., 2015). Some studies have also shown that while words elicit a stronger N170 response than pseudowords in the control group, this difference is not observed in individuals with DD (Mahé et al., 2012; Maurer et al., 2005). Although these studies have examined DD adults, they predominantly focus on alphabetic scripts. Chinese characters, however, differ significantly from alphabetic scripts in both visual and linguistic characteristics. To our knowledge, whether and how neural tuning for print is impaired in DD adults remains unclear. To advance our understanding of the neural basis of difficulties in visual word reading across different writing systems, it is crucial to determine whether neural tuning for print is universally impaired or specific to particular writing systems in individuals with reading difficulties.

**Figure 1:**
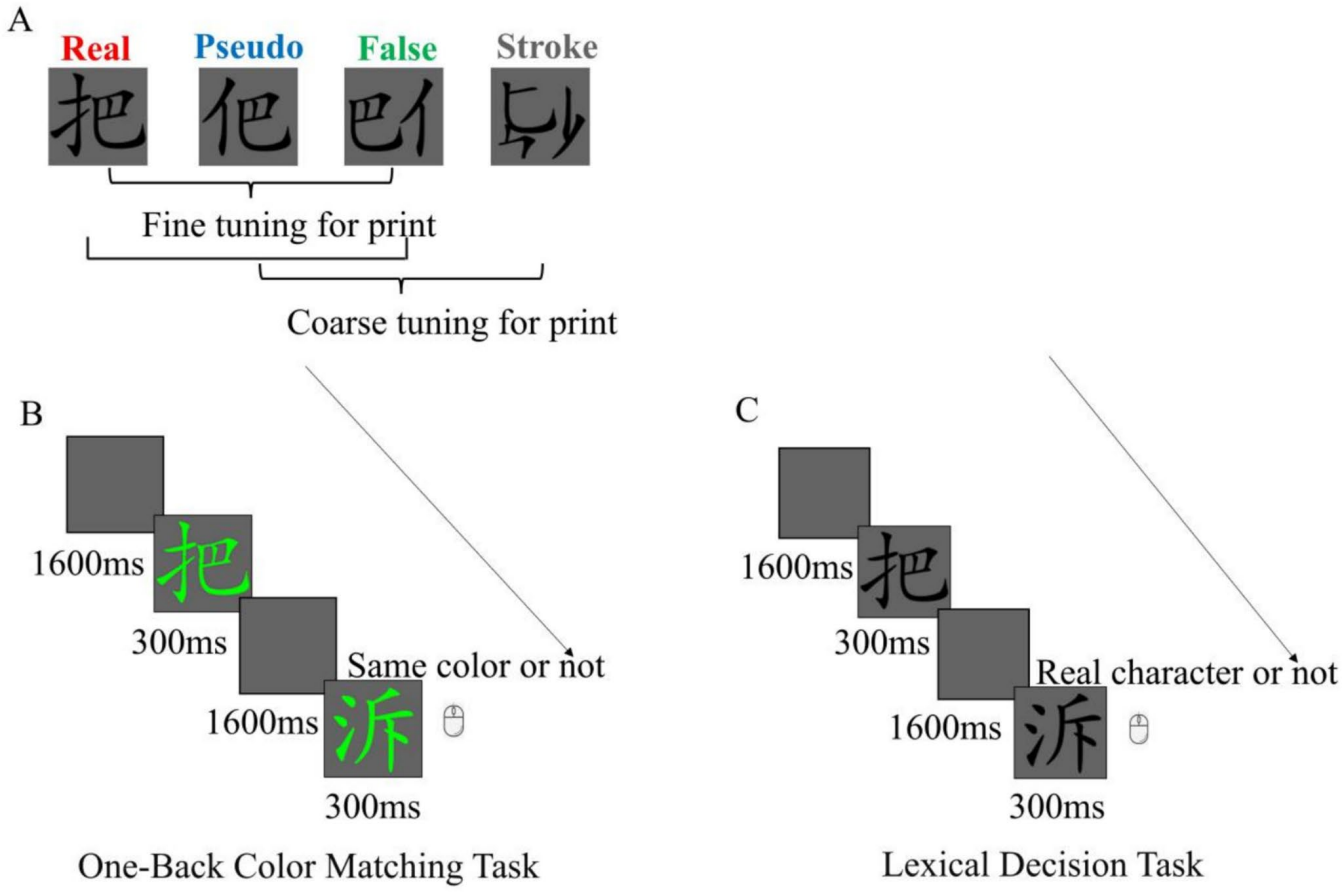
**(A)** Examples of stimuli and summary of the neural tuning for print including the coarse tuning (real, pseudo, and false characters vs. stroke combinations) and fine tuning (real vs. pseudo vs. false characters). **(B)** The schematic depiction of the color matching task in the EEG experiment. **(C)** The schematic depiction of the lexical decision task in the behavioral experiment.

The present study aimed to examine the characteristics of neural tuning for print in Chinese adults with low and high reading levels. To achieve this, we constructed four types of stimuli: real characters, pseudo characters, false characters, and stroke combinations (see Figure 1A for details). We focused on coarse tuning (real, pseudo, and false characters vs. stroke combinations) and fine tuning (real vs. pseudo vs. false characters). Following the procedures of previous studies (Liu et al., 2024; Zhao et al., 2014), we employed a grouping design to select two adult groups: a low-reading-level group and a high-reading-level group. Results in previous studies suggest that N170 responses to words especially Chinese characters may be prone to task modulation (see Zhao et al., 2012 for more discussion). To minimize the impact of task-related linguistic processing, we used a content-irrelevant color-matching task in the ERP experiment (see Figure 1B). This task has been widely used for examining the neural tuning for print (Huang et al., 2022; Xue et al., 2023; Zhao et al., 2014, 2019). To gain further insights into how participants classified these stimuli, we conducted a behavioral experiment with an explicit lexical decision task after the ERP experiment (Zhao et al., 2019).

Previous studies on individuals with alphabetic dyslexia have found that coarse tuning improves during reading development (Maurer et al., 2011). Recent research indicates that Chinese children with developmental dyslexia (DD), like typically developing (TD) children, exhibit coarse tuning for print (Xue et al., 2023). Based on these findings, we expected that Chinese adults with low reading levels would demonstrate reliable coarse tuning, as reflected in N170 selectivity for real, pseudo, and false characters relative to stroke combinations. However, previous studies have shown that fine tuning for print remains persistently impaired in individuals with alphabetic dyslexia (Mahé et al., 2012, 2013). Chinese children with DD have also shown no reliable fine tuning for print, even after 3 years of reading instruction (Xue et al., 2023). If fine neural tuning impairment is universally present across different writing systems, we would expect Chinese adults with low reading levels to exhibit no reliable fine tuning for print, as indicated by the absence of significant N170 differences among real, pseudo, and false characters.

## Method

### Participants

The required number of participants for this study was determined using a power analysis conducted with G*Power software (Faul et al., 2007). A total sample size of 24 participants (12 per group) was deemed necessary, with a power (1 − β) of 0.95, an alpha level of 0.05, and an effect size of 0.25. Aligned with previous study (Liu et al., 2024), we prioritized a high power of 0.95 to minimize the risk of type II error. Initially, 308 college students were screened, all of whom were right-handed native Chinese speakers with normal or corrected-to-normal visual acuity and no color vision defects. Reading ability was assessed using the Three-Minute Reading Test (Lei et al., 2010) and the Chain Test (Xia et al., 2018; Zou et al., 2012). Details of these tests are provided in the following section. Screening criteria, consistent with previous studies (Ding et al., 2016; Fu et al., 2019; Ho et al., 2004; Xue et al., 2023), included scoring below one standard deviation from the mean on the Three-Minute Reading Test and having at least one subtest score below one standard deviation from the mean on the Chain Test to identify the low-reading-level group. Conversely, the high-reading-level group was defined by a score on the Three-Minute Reading Test above one standard deviation from the mean and no subtest scores below one standard deviation from the mean on the Chain Test. Of the 39 individuals who met these criteria and agreed to participate, three were excluded due to noise in their EEG recordings, resulting in 20 participants in the high-reading-level group (M_age = 20.47, SD_age = 0.77) and 16 participants in the low-reading-level group (M_age = 19.44, SD_age = 0.81). The high- and low-reading level groups were the two extremes of the best and poorest readers of the database, respectively.

### Testing tools

#### Three-minute reading test

The Three-Minute Reading Test consists of 100 items, in which participants read sentences and determine their accuracy (e.g., “太阳从西边升起” [The sun rises from the west]). Participants are instructed to complete as many items as possible within a three-minute time frame. The difficulty of the items increases as the numbers progress, with longer sentences and higher difficulty levels. This design aims to prevent ceiling effects in adult reading assessments (Table 1).

**Table 1:** Mean age, Three-Minute Reading Test score and cognitive test score.

	High-reading-level (*n* = 20)		Low-reading-level (*n* = 16)			
	Mean	SD	Mean	SD	*t*	*p*
Age (years)	20.47	0.77	19.44	0.81	4.037	<0.001
Three-Minute reading	846.53	104.81	382.08	43.41	17.984	<0.001
Figure cancelation	52.55	8.03	46.50	8.79	2.155	0.038
Onset judgment	42.45	6.92	32.31	6.05	4.612	<0.001
Non-character recognition	49.60	6.41	38.00	7.67	4.945	<0.001
Tone	35.85	5.30	28.38	8.15	3.322	0.002
Word chain	48.30	7.05	32.50	5.16	7.492	<0.001
Animal word	43.75	4.28	35.44	4.70	5.543	<0.001
Pseudo-homophone detection	33.55	4.84	21.94	3.38	8.135	<0.001
Ambiguous sentence cross-out	30.30	9.04	14.19	7.83	5.631	<0.001

#### Chain test

The Chain Test is a cognitive assessment tool. The reading scores were adjusted by subtracting the number of false-alarm items from the number of hit items.

##### Figure-cross-out

This test was used as a baseline to assess general cognitive processing. Participants were instructed to find symbols composed of two S shapes (e.g., “§”) as quickly as possible within 30 s and mark them with a diagonal line. The test included 100 target symbols and 208 non-target symbols of other styles, randomly arranged in a 22 × 14 matrix. Prior to the timed test, participants completed a practice test consisting of 28 items to ensure they understood the experimental instructions.

##### Onset judgment

This test was designed to assess the mapping from orthography to phonology. Participants were instructed to find and cross out characters whose pronunciation began with the onset /b/ (e.g., “百” [bǎi]) within 80 s, regardless of tone. The test included 100 target characters starting with /b/ and 208 non-target characters randomly arranged in a 22 × 14 matrix. Prior to the timed test, participants completed a practice test consisting of 28 items to ensure they understood the experimental instructions.

##### Nonword cross-out judgment

This test was used to assess orthographic processing skills. Participants were instructed to identify and cross out non-characters as quickly as possible within 40 s. For example, “
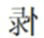
” is a non-character; although both radicals are correct, the radical “
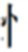
” is misplaced on the right side, preventing the combination from forming a valid character. Even if the radical “录” were in the correct position, the two radicals together would not form a valid character. Thus, all non-characters in this task were ill-formed and structurally incorrect. The test included 100 target non-characters and 208 non-target characters randomly arranged in a 22 × 14 matrix. Prior to the timed test, participants completed a practice test consisting of 28 items to ensure they understood the experimental instructions.

##### Tone test

This test was used to measure phonological awareness. Participants were instructed to cross out characters with the fourth tone (e.g., “戏” [xì], play) within 90 s, regardless of their initial consonants and final vowels. The test included 100 target characters with the fourth tone and 208 non-target characters with other tones (e.g., “习” [xí], study), randomly arranged in a 22 × 14 matrix. Prior to the timed test, participants completed a practice test consisting of 28 items to ensure they understood the experimental instructions.

##### Word chains

This test was used to measure the ability to identify the word boundaries. Participants were required to scan words presented in a continuous line of print without inter-word spaces (e.g., “书车老师”). They were instructed that each of the 88 clusters consisted of three words and that their task was to identify the word boundaries within each cluster by using slashes within 80 s (e.g., “书(book)/车(car)/老师(teacher)”). Before the formal timed test, participants completed a practice test consisting of 8 clusters of three words each to ensure they understood the experimental instructions.

##### Animal-word cross-out

This task was designed to assess the mapping from orthography to semantics. Participants were instructed to identify and cross out words representing animals (e.g., “飞鸟”/fei1niao3/, flying bird) as quickly as possible within 50 s. The test included 100 target characters and 208 non-target characters randomly arranged in a 22 × 14 matrix. Prior to the timed test, participants completed a practice test consisting of 28 items to ensure they understood the experimental instructions.

##### Pseudo-homophone discrimination

This test was designed to assess the mapping from orthography to phonology and semantics (Mental lexicon accessing). Participants were instructed to identify and cross out pseudo-homophone words that do not exist in Chinese (e.g., “平果” /píng guoˇ/) but are pronounced the same as real words (e.g., “苹果” /píng guoˇ/, apple) within 70 s. The test included 100 target characters and 208 non-target characters, which were randomly arranged in a 22 × 14 matrix. Prior to the formal timed test, participants completed a practice test consisting of 28 items to ensure they understood the experimental instructions.

##### Ambiguous sentence judgment

This task was administered to evaluate the processing of syntax and meaning during reading. Participants were instructed to quickly determine whether sentences were ambiguous within 180 s. For ambiguous sentences, they were to cross out the word “有” (yes); for unambiguous sentences, they were to cross out the word “无” (no) (e.g., “扮演的是一个美国犹他州著名黑人喜剧演员 有 无,” played by a famous African American comedian from Utah, yes, no). Before the formal timed test, participants completed a practice test consisting of four items to ensure they understood the experimental instructions. The total number of sentences was 69.

### Materials

Consistent with the research protocols of Zhao, the current study used Chinese characters containing two radicals arranged horizontally (Zhao et al., 2019). According to the Modern Chinese Frequency Dictionary (1985), the word frequency typically ranges from 300 to 600 per million. We employed four types of character stimuli: real, pseudo, false, and stroke combinations (see Figure 1A). Pseudo and false characters were constructed using the same set of radicals as real characters. In pseudo characters, the radicals were positioned legally, while in false characters, the radicals were placed in illegal positions. Stroke combinations were created by disrupting the strokes of false characters while preserving their left and right structures. This allowed us to control for visual complexity and structure. Abstract symbols, which were used in previous studies (Maurer et al., 2005, 2006), may not share the same visual properties as Chinese characters, and potentially involve confounds related to low-level visual features. Therefore, we did not use the abstract symbols as low-level visual control in our study.

Each stimulus type consisted of 36 stimuli, with 6 serving as targets, following the procedure used in previous studies, which employed a target-to-non-target ratio of approximately 1:5 (Huang et al., 2022; Xue et al., 2023; Zhao et al., 2019). This ratio ensures an adequate number of target trials for statistical analysis while maintaining the rarity of targets, which is crucial for maintaining attention and engagement throughout the experiment. Each stimulus appeared in one of three colors: green, red, or yellow, with an equal distribution of colors across all stimulus types in the one-back color matching task. Additionally, an equivalent set of stimuli in black was used in the lexical decision task.

### Procedure

#### One-back color matching task

The one-back color matching task was programmed using E-Prime 2.0. The four types of stimuli were mixed and presented in random order, with the constraint that consecutive stimuli of the same type were not allowed. Each stimulus appeared in the center of the screen against a gray background. During each trial, the stimulus was displayed for 300 ms. The inter-stimulus interval (ISI) was randomly selected from 1,450 ms, 1,525 ms, 1,600 ms, 1,675 ms, or 1,750 ms. Participants were required to press any key on the mouse with either the left or right index finger when two consecutive stimuli of the same color appeared (see Figure 1B). Response keys were balanced across participants. To obtain a sufficient number of averaged event-related potentials (ERPs), each trial was presented three times (for similar experimental manipulations, see Ca and Zhang, 2011; Lin et al., 2011; Maurer et al., 2008; Zhao et al., 2012, 2019). The experiment consisted of four blocks, each comprising 108 trials (90 non-target trials and 18 target trials).

#### Lexical decision task

The lexical decision task was programmed using Python and PsychoPy-2022 (see Figure 1C). There were four types of stimuli, each with 36 trials. Each block consisted of 144 trials, with all stimuli presented three times. Stimuli were presented randomly, with the constraint that the same type of stimulus could not appear consecutively. Each stimulus was displayed for 300 ms, and the inter-stimulus interval (ISI) was randomly selected from 1,450 ms, 1,525 ms, 1,600 ms, 1,675 ms, or 1,750 ms. Participants were instructed to judge whether the presented stimulus was a real Chinese character by pressing either the “F” or “J” key as accurately and quickly as possible. Response keys were balanced across participants.

### Data collection and statistical analysis

The EEG signal was recorded using a DC amplifier system (BrainAmp ExG, Brain Products GmbH, Gilching, Germany) with 64 Ag/AgCl electrodes secured in an elastic cap according to the extended international 10–20 system. EEG data were recorded using BrainVision Recorder software (Brain Products GmbH, Germany). The ground electrode was positioned at AFz, and an electrode placed between Cz and CPz served as the online reference. The data were re-referenced to a common average reference across all electrodes during later analysis, excluding the electrooculogram (EOG) electrodes. Both vertical and horizontal EOG signals were recorded for eye-blink correction. Electrode impedances were kept below 5 kΩ. EEG and EOG signals were continuously recorded, amplified with a bandpass filter from 0.1 to 100 Hz, and sampled at 1,000 Hz.

Offline data processing included band-pass filtering with a low-pass cutoff at 30 Hz and a high-pass cutoff at 0.1 Hz. Each recording epoch was manually inspected for artifacts before averaging. Eye movement artifacts were corrected offline using an independent component analysis (ICA) procedure. Trials were segmented and baseline-corrected with a 100 ms prestimulus period, and the poststimulus period lasted 800 ms. For further analyses, only non-target trials with no false positive (target) responses were included. Artifacts exceeding ±100 μV were automatically rejected prior to averaging. In this study, as in many others, the N170 component was used to assess visual object processing. Occipitotemporal electrodes are recognized as the most representative for measuring the N170 component (Schlaggar and McCandliss, 2007; Zhao et al., 2019). Based on this convention, we selected five pairs of occipitotemporal channels: O1/O2, P7/P8, TP9/TP10, P5/P6, and PO7/PO8. A global field power method was used to determine the N170 time window for each group (Maurer et al., 2005). In the statistical analysis, N170 amplitudes were averaged separately for each hemisphere (i.e., left hemisphere: O1/P7/P5/TP9/PO7; right hemisphere: O2/P8/P6/TP10/PO8).

Following the EEG experiment, participants were administered a lexical decision task. In accordance with prior research (Zhao et al., 2019), this task was employed to evaluate participants’ efficiency in categorizing four types of stimuli.

## Results

### Results in the lexical decision task

**Figure 2:**
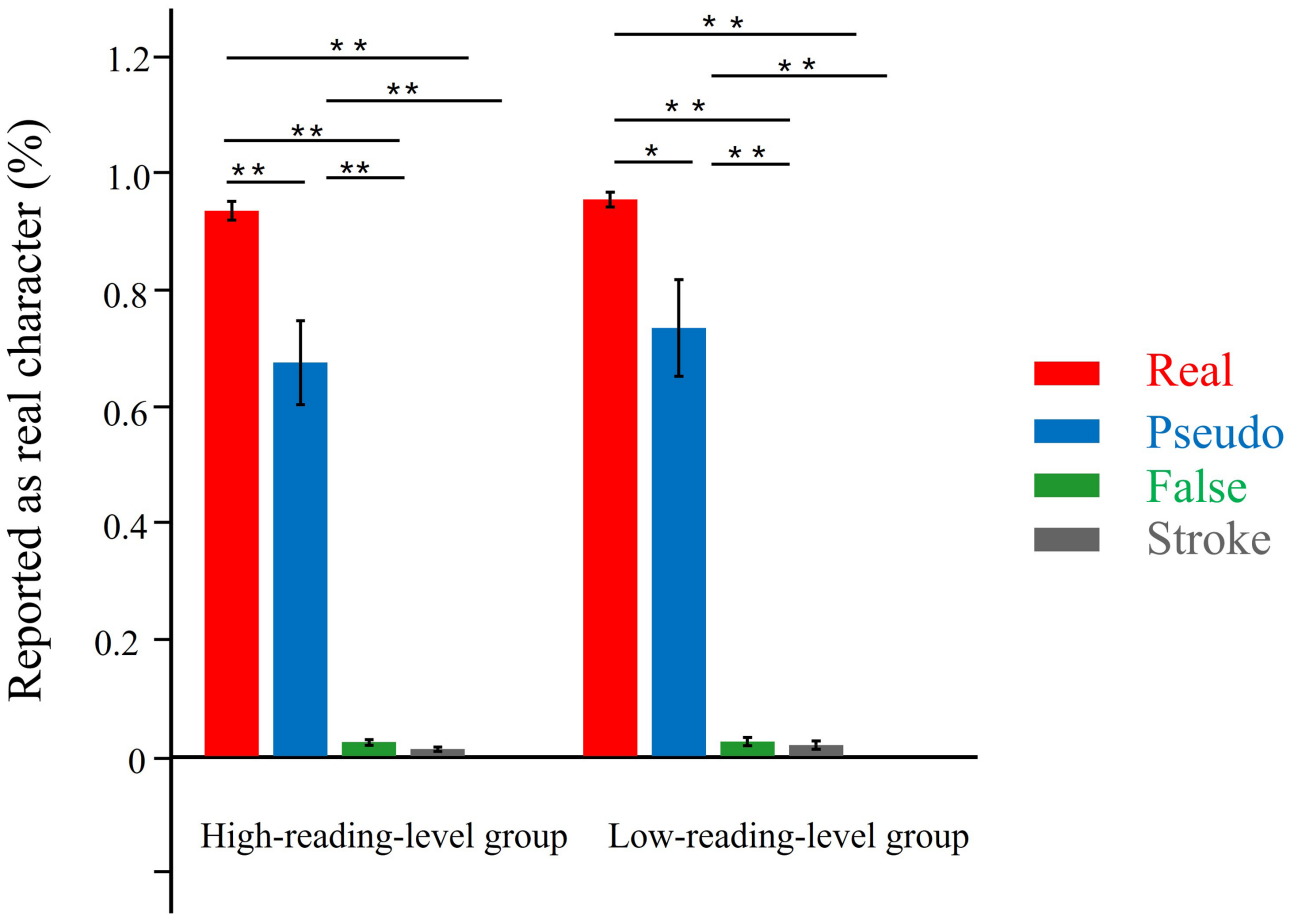
The rate in reporting a stimulus as a real character in the high and low reading level groups in the lexical decision task. **p < 0.01, *p < 0.05.

Figure 2 illustrates the rate at which a stimulus is reported as a real character by adults with low and high reading levels. The data were analyzed using a two-way ANOVA, with character-response rate (i.e., the proportion of stimuli reported as real characters) as the dependent variable, the stimulus type and reading group as the independent variables. Trials in which the reaction time for each participant exceeded ±3 standard deviations were excluded from the calculation of character-response rate. The results indicated a significant main effect of stimulus type, *F* (3, 99) = 305.054, *p* < 0.001, *η*^2^ = 0.902. The other main effect was not significant. No significant interactions were observed between stimulus type and reading group, *F* (3, 99) = 0.231, *p* = 0.654, *η*^2^ = 0.007.

To test the research hypothesis, we conducted additional planned analyses using Tukey’s HSD correction. As shown in Figure 2, the results indicated that, regardless of reading level, the rate at which the four stimuli (real characters, pseudo characters, false characters and stroke combinations) were reported as real characters decreased progressively, with significant differences observed in all pairwise comparisons of the four stimuli (all *p*-values < 0.05), except for the comparison between false characters and stroke combinations (*p* > 0.139). Additionally, no significant differences in the rate of character response to any of the four stimuli was observed between the high-reading-level and low-reading-level groups (all *p*-values > 0.05).

### N170 results

**Figure 3:**
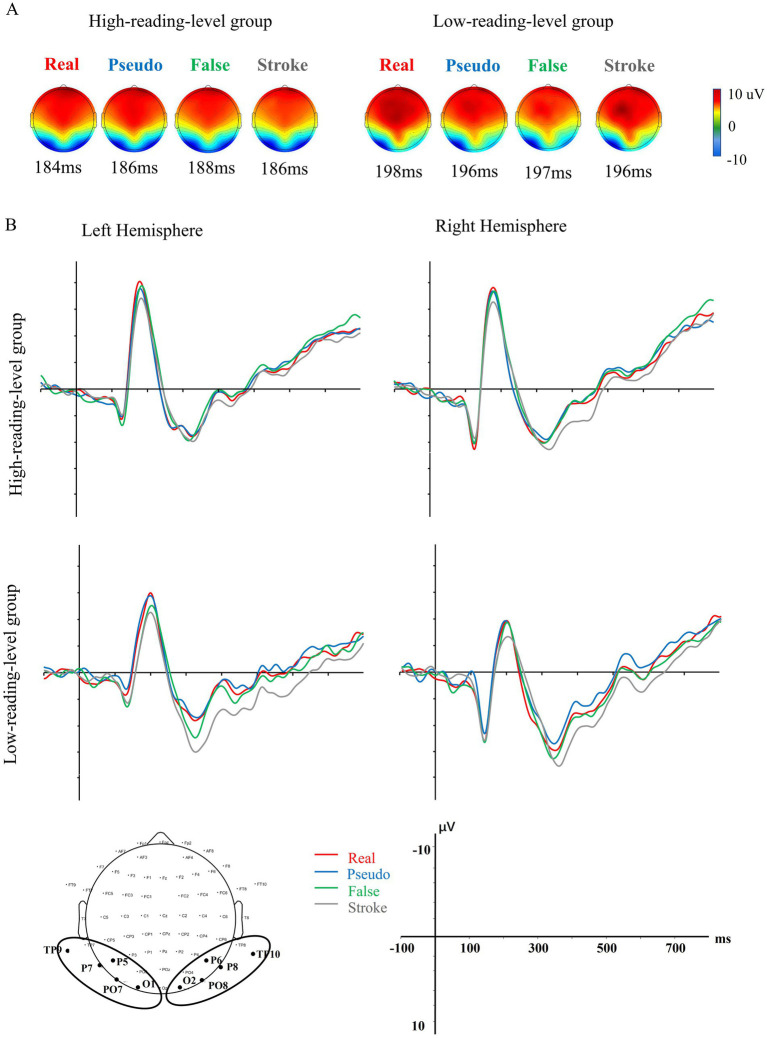
**(A)** Topographic maps of 4 stimulus types at N1 peaks in the low and high reading level groups. **(B)** Grand averaged ERP waves of both ROI in the two groups.

Figure 3 illustrates the topographic maps and the occipitotemporal ROI ERP waveforms for the four stimulus types, separately for adults with low and high reading levels. A robust N170 component was observed for each stimulus type in both groups.

Data were analyzed using a repeated measures ANOVA to assess the average N170 amplitudes in each hemisphere. Additionally, a three-way ANOVA was conducted with two within-subject factors—stimulus type (real, pseudo, false, stroke) and lateralization (left and right)—and one between-subject factor: reading group (high-reading-level group and low-reading-level group).

The results indicated that the main effects of stimulus type (*F*(3, 102) = 10.743, *p* < 0.001, *η*^2^ = 0.326) and group (*F*(1, 34) = 7.736, *p* < 0.01, *η*^2^ = 0.185) were significant. However, the main effect of lateralization was not significant (*F* < 1). The results also revealed non-significant interactions. Specifically, there were no significant interactions between stimulus type and group (*F*(3, 102) = 1.595, *p* = 0.204, *η*^2^ = 0.045), stimulus type and lateralization (*F*(3, 102) = 0.565, *p* = 0.642, *η*^2^ = 0.050), or among stimulus type, lateralization, and group (*F*(3, 102) = 0.769, *p* = 0.494, *η*^2^ = 0.022). To test the research hypothesis, planned analyses were conducted. Specifically, a planned *post hoc* simple effects analysis was performed on the three-way interaction using Tukey’s HSD correction. As shown in Figure 3, the N170 amplitude for real and pseudo characters was more negative compared to stroke combinations in the low-reading-level group (*p* < 0.05). Similarly, in the high-reading-level group, N170 amplitudes were stronger for real characters compared to stroke combinations (*p* = 0.032). In summary, these results suggest that coarse tuning may be intact in adults with both high and low reading levels.

**Figure 4:**
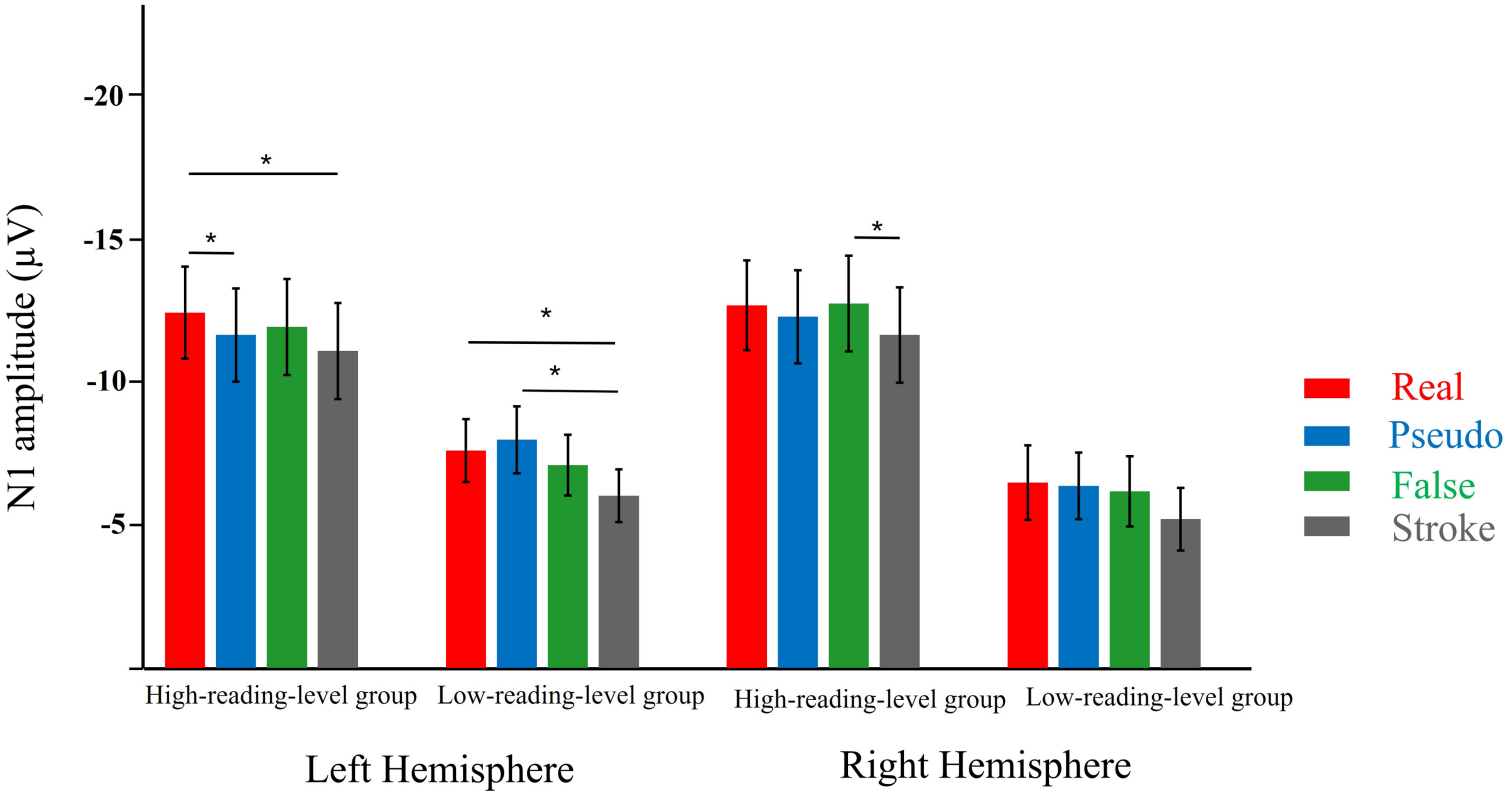
Mean N170 amplitudes for the four stimulus types in the left and right ROIs in low and high read level groups. *p < 0.05. Error bars represent standard errors.

The fine-tuning for print primarily involved the N170 response to three types of orthographic stimuli (i.e., real, pseudo, false characters). Therefore, a 3 × 2 × 2 three-way ANOVA was conducted, with stimulus type (real, pseudo, false) and lateralization (left and right) as within-subject factors and reading group (high-reading-level group and low-reading-level group) as a between-subject factor. The results showed no significant interactions across all conditions. Specifically, there were no significant interactions between stimulus type and group (*F*(2, 33) = 2.113, *p* = 0.133, *η*^2^ = 0.059), stimulus type and lateralization (*F*(2, 33) = 0.777, *p* = 0.468, *η*^2^ = 0.045), or among stimulus type, lateralization, and group (*F*(3, 102) = 0.904, *p* = 0.392, *η*^2^ = 0.026). To test the research hypothesis, planned analyses were performed. Specifically, a planned post hoc simple effects analysis was conducted on the three-way interaction using Tukey’s HSD correction. As shown in Figure 4, in the high-reading-level group, the N170 response to false characters was greater than to pseudo characters (*p* = 0.817), and the response to real characters was significantly stronger than that to pseudo characters (*p* = 0.041) over the left hemisphere. In contrast, in the low-reading-level group, there were no significant N170 differences among the three types of stimuli (all *p*-values > 0.215). Over the right hemisphere, no significant differences in N170 were detected among the stimulus types in either reading level group (all *p*-values > 0.470). In summary, these results suggest that fine-tuning may be impaired in adults with low reading levels.

The N170 peak latency means and standard deviations for the four stimuli across adults with low and high reading levels are reported in Table 2. Data were analyzed using a three-way ANOVA with two within-subject factors—stimulus type (real, pseudo, false, stroke) and lateralization (left, right)—and one between-subject factor: reading group (high-reading-level, low-reading-level). The analysis revealed a significant main effect of lateralization (*F*(1, 34) = 5.435, *p* = 0.026, *η*^2^ = 0.138). However, the main effects of stimulus type (*F*(3, 102) = 2.486, *p* = 0.065, *η*^2^ = 0.068) and group (*F*(1, 34) = 3.364, *p* = 0.071, *η*^2^ = 0.092) were not significant. A significant interaction was found between stimulus type and lateralization (*F*(3, 32) = 3.198, *p* = 0.036, *η*^2^ = 0.231), while the interactions between stimulus type and group (*F*(3, 102) = 0.633, *p* = 0.595, *η*^2^ = 0.018), and among stimulus type, lateralization, and group (*F*(3, 102) = 0.463, *p* = 0.653, *η*^2^ = 0.013) were non-significant. Further analysis showed that in the high-reading-level group, N170 peak latency for false characters was significantly longer in the left hemisphere than in the right hemisphere. Similarly, in the low-reading-level group, N170 peak latency for both real and false characters was significantly longer in the left hemisphere than in the right hemisphere.

**Table 2:** The N170 latency (ms) for the four stimulus types in two groups.

	High-reading-level		Low-reading-level	
	LH	RH	LH	RH
Real	184 (18)	182 (20)	198 (15)	191 (17)
Pseudo	186 (16)	182 (22)	196 (16)	191 (17)
False	188 (17)	181 (22)	197 (18)	191 (18)
Stroke	186 (20)	185 (22)	198 (16)	196 (20)

Standard errors of means are given in parentheses.

## Discussion

The purpose of this study was to examine the characteristics of neural tuning for print, as reflected in N170 selectivity for words compared to other visual control stimuli, in adults with varying reading levels. We observed both low and high reading level adults exhibited a stronger N170 response to real, pseudo, or false characters compared to stroke combinations. However, adults with high reading levels showed a stronger N170 response to real characters than to pseudo characters, while adults with low reading levels showed no significant difference between real and pseudo characters. Overall, these results indicate that fine-tuning for print may be selectively impaired in adults with low reading levels.

At the behavioral level, adults, regardless of reading level, efficiently rejected false characters and stroke combinations as real characters when judging whether a stimulus was a genuine Chinese character. However, accuracy in identifying pseudo characters as real characters was relatively low. These results suggest that the pattern of classifying stimuli during the lexical decision task was similar between the two groups. Our findings align with those of previous studies (Mahé et al., 2012; Shaul, 2013; Shaul et al., 2012; Taroyan and Nicolson, 2009), indicating that the pattern of lexically classifying visual stimuli in adults with low reading levels was similar to that in adults with high reading levels.

At the neural level, our primary finding was that coarse tuning for print was intact in Chinese adults with low reading levels. Results indicated that both low and high reading level adults exhibited stronger N1 responses to real, pseudo, or false characters compared to stroke combinations. In contrast, previous studies have found that, unlike typically developing (TD) adult readers, adults with developmental dyslexia (DD) do not show significant N1 amplitude differences between letter strings and symbol strings, suggesting a deficit in coarse tuning for print among alphabetic DD adults (Helenius, 1999; Mahé et al., 2012, 2013; Shaul et al., 2012). A plausible explanation for this discrepancy is the significant linguistic differences between Chinese characters and alphabetic words. In alphabetic languages, letters and other orthographic forms systematically map onto sounds. Research on alphabetic readers indicates that coarse tuning for print emerges quickly in preschool children once grapheme-phoneme correspondences are acquired (Brem et al., 2010). Even after learning to read, individuals with DD often exhibit impaired phonological decoding skills (Maurer et al., 2007, 2008), making coarse tuning more susceptible to impairment in alphabetic DD adults. In contrast, the visual forms in the Chinese writing system map onto phonology relatively arbitrarily (Chen and Juola, 1982). Orthography, rather than phonology, primarily drives N170 selectivity for print in Chinese (Lin et al., 2011). Furthermore, coarse tuning for print in this context may reflect processes related to coarse orthographic encoding (Brem et al., 2010; Eberhard-Moscicka et al., 2016; Maurer et al., 2006; Zhao et al., 2019). With reading practice, Chinese adults refine their knowledge of orthographic rules, enabling them to distinguish characters from stroke combinations. Therefore, coarse tuning for print in Chinese adults with low reading levels appears to be intact. Additionally, differences in task demands may influence the results. The present study employed a non-linguistic color-matching task, whereas previous studies often used linguistic tasks, such as lexical decision tasks (Apel, 2011). In linguistic tasks, coarse tuning for print may be more vulnerable and reliant on phonological processing (Zhao et al., 2012).

Our second main finding was that fine-tuning for print was impaired in Chinese adults with low reading levels. Specifically, adults with high reading levels exhibited a stronger N170 response to real characters compared to pseudo characters, whereas adults with low reading levels showed no significant N170 difference between real and pseudo characters. This pattern is consistent with previous research indicating impaired fine-tuning for print in alphabetic developmental dyslexia (DD) adults. For example, Araújo and Mahé reported no differences between pseudowords and unpronounceable letter strings in DD adults (Araújo et al., 2015; Mahé et al., 2013). Similar to our findings, other studies have shown that while words elicit a stronger N170 response than pseudowords in control groups, this effect is not observed in DD adults (Mahé et al., 2012; Maurer et al., 2005). Given that the real characters used in our study were high-frequency Chinese characters, our results align with studies reporting that words elicit a more negative N1 response than pseudowords in adults (Mahé et al., 2012; Maurer et al., 2005; Savill and Thierry, 2012). One possible explanation for the impaired fine-tuning in DD adults is that the ability to differentiate between real and pseudo characters relies on lexical access. The implicit task used in the present study (e.g., the color-matching task) did not require explicit linguistic processing (Zhao et al., 2012). Adults with DD may have difficulty accessing the mental lexicon and finely distinguishing between real and pseudo characters. This is consistent with previous findings suggesting that efficient lexical access and differentiation between familiar and unfamiliar words are crucial for reading proficiency (Aitchison, 2012; Perfetti, 2007). Therefore, we did not find robust fine-tuning for print in DD adults. As noted earlier, impaired fine-tuning was observed in adults with low reading levels.

More broadly, from a developmental perspective, our findings extend previous research on children with developmental dyslexia (DD). Extensive studies have explored neural tuning for print in DD children with alphabetic scripts, revealing a developmental delay rather than a persistent deficit in coarse tuning for print (Maurer et al., 2011). However, deficits in fine-tuning for print persist throughout reading development (Araújo et al., 2015; Bakos et al., 2018; Mahé et al., 2012). Recently, Xue and colleagues found intact coarse neural tuning but impaired fine-tuning for print in Chinese DD children (Xue et al., 2023). As discussed above, our results further suggest that Chinese adults with low reading levels also exhibit a selective impairment in fine-tuning for print. Together with previous studies, these findings indicate that fine-tuning for print may remain impaired despite extensive reading practice. These results also offer neurophysiological markers for objective electrophysiological measures of adult reading ability.

### Limitations

We would like to highlight several limitations of this study. First, defining adult dyslexia in Chinese is challenging because, unlike in alphabetic scripts where dyslexia is diagnosed and documented in childhood, there is no standardized approach for Chinese adults. Consequently, we examined adults with low reading levels. Future research should refine the diagnosis of adult dyslexia in Chinese or implement early diagnosis and documentation to allow for a more precise investigation into the neural basis of visual word processing deficits in Chinese adult dyslexia. Second, we used a color-matching task that did not require explicit linguistic processing, leaving it unclear whether neural tuning for print is impaired in Chinese adults with low reading ability during language-related tasks. Future studies incorporating language-related tasks will provide additional evidence to address this issue. Third, our sample was limited to college students. Future research should include a broader range of participants to verify our findings.

## Conclusion

We observed that adults, regardless of reading level, exhibited increased N170 responses to real characters compared to stroke combinations. However, adults with high reading levels had significantly higher N170 responses to real characters than to pseudo-characters, while adults with low reading levels demonstrated less pronounced N170 differences between these two types of stimuli. These results suggest that Chinese adults with low reading levels have selective impairments in fine neural tuning for print. Furthermore, these findings offer objective electrophysiological markers for assessing reading ability in Chinese adult readers.

## Data Availability

The data used to support the findings of this study are available from the corresponding author upon request.
